# Burkitt Lymphoma Presenting as Ileocolic Intussusception in an Adult

**DOI:** 10.4236/ojbd.2023.134014

**Published:** 2023-12-08

**Authors:** Carla Isabel Borré, Bethany Boyle, Kelsey Lynch, Anuradha Kanaparthi, Clifford Michael Csizmar, Daniel Philip Larson, Matthew Dain Braithwaite, Isla McKerrow Johnson, Thomas Elmer Witzig, Diego Armando Suarez

**Affiliations:** 1 Yale School of Public Health, Yale University, New Haven, USA; 2 Department of Medicine, Mayo Clinic, Rochester, USA; 3 Division of Hematopathology, Department of Laboratory Medicine and Pathology, Mayo Clinic, Rochester, USA; 4 Division of Hematology, Department of Medicine, Mayo Clinic, Rochester, USA; 5 Division of Community Internal Medicine, Department of Medicine, Mayo Clinic, Rochester, USA

**Keywords:** Burkitt Lymphoma, Adult Intussusception, Prognostication

## Abstract

Adult intussusception is rare, highly associated with a malignant lead point, and often requires emergent surgical management. We report the case of a 44-year-old male who presented with generalized abdominal pain and was found to have early ileocolic intussusception secondary to a large ileocecal mass. Biopsies of the mass and an enlarged cardiophrenic lymph node, as well as pleural fluid cytology were all consistent with Burkitt lymphoma (BL). Curiously, the patient’s abdominal exam was reassuring, and the intussusception and malignant bowel obstruction resolved over 36 hours with conservative management alone. With a Burkitt lymphoma international prognostic index (BL-IPI) score of 2, the patient proceeded to treatment with combination chemoimmunotherapy and attained a complete response after four cycles. There was no bowel perforation or recurrent intussusception throughout treatment. Thus, this report marks the first reported case of adult BL-associated intussusception to resolve with non-invasive management and establishes a precedent for conservative management in select patients.

## 1. Introduction

Numerous studies have established a correlation between bowel intussusception in adults and malignant lead points, including gastrointestinal carcinomas, non-Hodgkin lymphomas, and metastatic tumors [1] [2]. Though common in the pediatric population, Burkitt lymphoma (BL) is a rare cause of intussusception in adults, and little is known about the prognosis and survival of these patients. In this report, we present a unique case of BL-associated ileocolic intussusception in an adult patient that resolved with conservative management. Thus, this case establishes a precedent for the non-invasive management of BL-associated intussusception in very select patients.

## 2. Case Presentation

A 44-year-old, previously healthy male presented to a local emergency department (ED) with a four-week history of poorly localized abdominal pain. He endorsed nausea and non-bloody emesis with associated bloating, anorexia, and 20 pounds of unintentional weight loss over the preceding month. Abdominal imaging via computed tomography (CT) revealed a distal small bowel obstruction, and the patient was transferred to our ED for further management.

**Figure 1.**
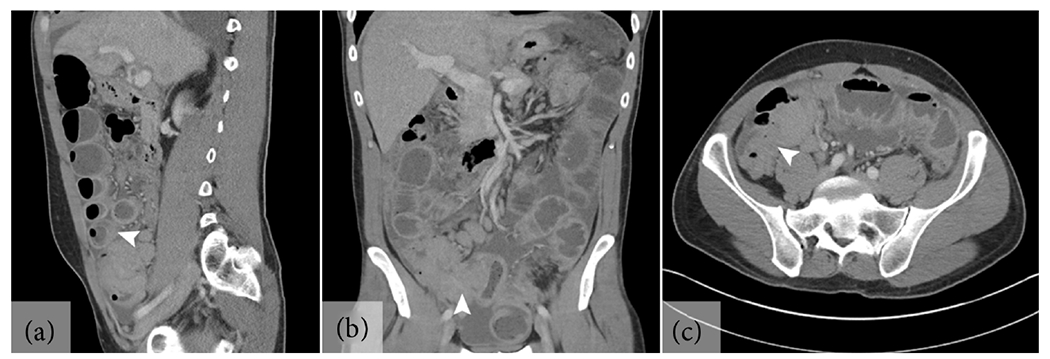
Computed tomography imaging of early ileocolic intussusception. (a) Sagittal view showing dilated bowel with air fluid levels and an early distal target sign (arrowhead). (b) Coronal view showing the transition point centered on an ileocecal mass (arrowhead). (c) Axial view of the ileocecal mass (arrowhead) and air-fluid levels.

Upon arrival, the patient’s vital signs were stable with blood pressure 148/75 mmHg, pulse 89 beats/min, respiratory rate 18 breaths/min, SpO_2_ 96% breathing ambient air, and temperature 36.6°C. On examination, there was no abdominal tenderness to palpation, distention, or peritoneal signs. Laboratory findings were notable for a normal complete blood count including differential, creatinine 1.1 mg/dL with unknown baseline, a mild anion gap metabolic acidosis with a bicarbonate of 21 mmol/L and an anion gap of 17, and elevated liver enzymes with alanine aminotransferase (ALT) 48 U/L (reference range [ref.] 7 - 55), aspartate aminotransferase (AST) 58 U/L (ref. 8 - 48), and alkaline phosphatase (ALP) 72 U/L (ref. 40 - 129). Institutional interpretation of the outside CT indicated small bowel obstruction with early ileocolic intussusception at theileocecal valve with pathological lead point (Figure 1). Diffuse lymphadenopathy above and below the diaphragm, bilateral pleural effusions, and ascites with early peritoneal carcinomatosis were also described. The patient was admitted to Internal Medicine for management of theintestinal obstruction.

**Figure 2.**
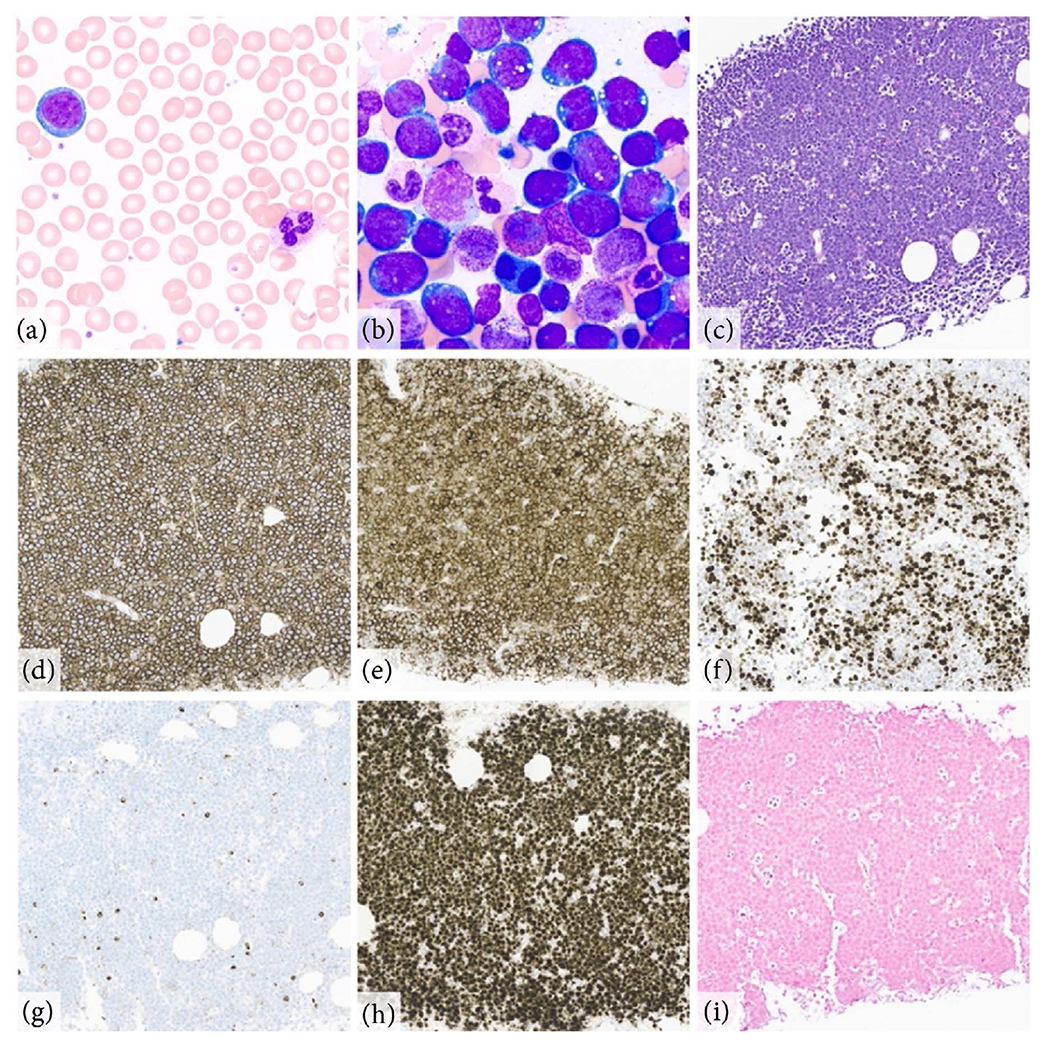
Histopathology consistent with Burkitt Lymphoma. Representative images of Wright Giemsa stained preparations (1000× magnification) from peripheral blood (a) and bone marrow aspirate (b) demonstrating an atypical aggressive-appearing lymphoid population with cytoplasmic vacuoles characteristic of Burkitt lymphoma. Biopsy of an affected cardiophrenic lymph node (100× magnification) shows a “starry sky” appearance by H&E (c). Immunohistochemistry reveals positive expression of CD20 (d), CD10 (e), BCL6 (f), negative expression of BCL2 (g), and overexpression of MYC (h). There was no expression of Epstein-Barr virus by *in situ* hybridization (i). These features support a diagnosis of Burkitt lymphoma.

He underwent bowel decompression with nasogastric tube placement and received intravenous (IV) fluids. In consultation with both Gastroenterology and Colorectal Surgery, a trial of conservative management (including close observation with serial abdominal examinations) was pursued given the patient’s reassuring abdominal exam. Fortunately, his bowel obstruction resolved within 36 hours as demonstrated by symptom improvement, passage of flatus and stool, and successful progression to a regular diet. Meanwhile, the patient underwent a comprehensive evaluation. Additional laboratory studies were significant for a rising serum creatinine to 1.56 mg/dL with serum potassium 4.8 mmol/L, phosphorus 4.8 mg/dL (ref. 2.5 - 4.5), uric acid 11 mg/dL (ref. 3.7 - 8.0), and lactate dehydrogenase (LDH) 640 U/L (ref. 122 - 222). A peripheral blood smear revealed rare atypical lymphocytes, with reflex flow cytometry demonstrating a corresponding monotypic CD10-positive B cell population. The patient’s evaluation for an underlying malignancy included thoracentesis with pleural fluid cytology, endoscopic sampling of the ileocolonic mass, and guided biopsy of the enlarged cardiophrenic lymph nodes. The patient remained clinically improved and was discharged with close follow up. Days later, the lymph node biopsy results demonstrated a high-grade B cell lymphoma most consistent with Burkitt lymphoma (Figure 2). Concurrent fluorescence in-situ hybridization (FISH) revealed a *MYC/IGH* fusion in approximately 91% of nuclei. There was no rearrangement of *BCL2* or *BCL6*. The pleural fluid and ileocolonic mass were involved by the same lymphoma cells.

Given the high risk of spontaneous tumor lysis syndrome (TLS) in Burkitt lymphoma and the patient’s recent acute kidney injury, he was readmitted to the Hospital Internal Medicine service for expedited evaluation and management. Re-admission laboratory results were notable for creatinine 1.46 mg/dL, potassium 5.4 mmol/L, phosphorus 5.1 mg/dL, LDH 1,057 U/L, and uric acid 17.1 mg/dL. The liver enzymes had also further elevated to ALT 155 U/L, AST 155 U/L, and ALP 143 U/L. The patient was treated for spontaneous TLS with IV fluids, rasburicase, and allopurinol, and his renal function slowly improved to a creatinine of 0.87 g/dL. Bone marrow biopsy revealed greater than 50% involvement by high grade B cell lymphoma (Figure 2). Fluorodeoxyglucose (FDG)-positron emission tomography (PET)/CT confirmed extensive nodal, mesenteric, and peritoneal involvement in addition to the large, intensely FDG-avid (maximum standardized uptake value [SUVmax] 14.5) ileocolonic mass. Lumbar puncture with cerebrospinal fluid (CSF) cytology and flow cytometry showed no evidence of central nervous system (CNS) disease. Human immunodeficiency (HIV) and chronic hepatitis serologies were negative.

**Figure 3.**
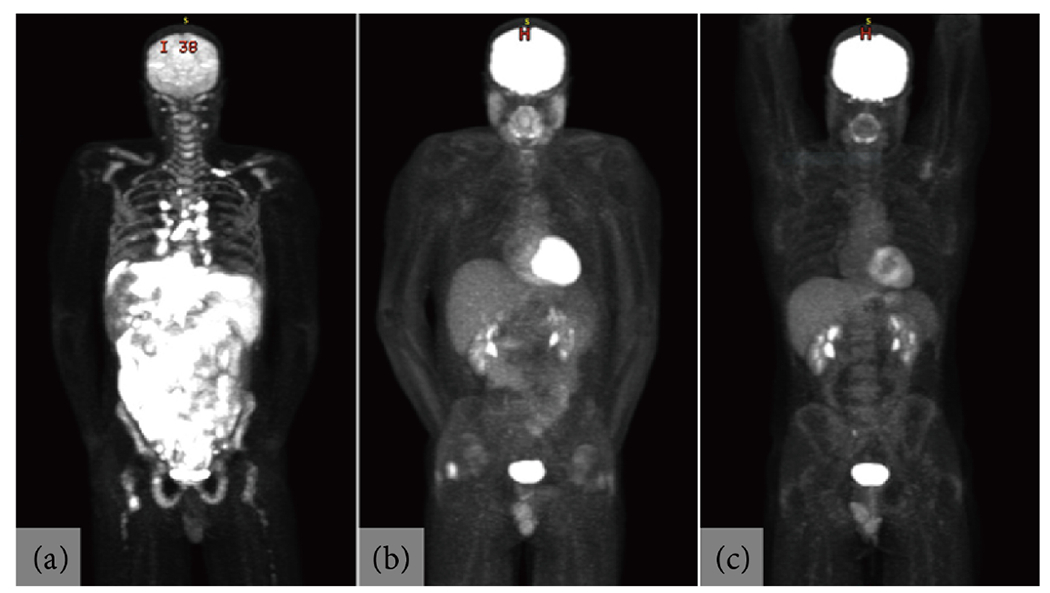
Staging, Interim, and Post-Treatment PET/CT Images. Representative images of PET/CT scans obtained (a) at the time of diagnosis; (b) after two cycles of R-CODOX-M/IVAC, demonstrating a near complete response; and (c) after all four cycles of therapy, demonstrating a complete response (Deauville score 1).

Thus, the patient was diagnosed with high-risk (by standard criteria), stage IV, Burkitt lymphoma. He was also considered high-risk by the recently-described Burkitt lymphoma international prognostic index (BL-IPI), meeting two criteria (age and LDH) to predict 3-year progression-free survival (PFS) and overall survival (OS) of 63% and 64%, respectively [3]. Thus, he was transferred to the inpatient Hematology service for four cycles of highly dose-intensive chemoimmunotherapy consisting of rituximab in combination with cyclophosphamide, doxorubicin, vincristine, and methotrexate (R-CODOX-M) alternating with ifosfamide, etoposide, and cytarabine (IVAC). Interim PET/CT showed a near complete response, and post-treatment PET/CT confirmed a complete response (Deauville score 1; Figure 3). The patient remained in complete remission at last follow up, 10 months after initial diagnosis.

## 3. Discussion

Burkitt lymphoma (BL) is a high-grade Bcell lymphoma genetically characterized by deregulated *MYC* oncogene expression due to chromosomal translocations that juxtapose *MYC* and an immunoglobulin locus, classically t(8; 14) (q24;q32) [4]. Historically, three epidemiologic variants of BL have been described: endemic, sporadic (non-endemic), and immunodeficiency-associated [5]. However, this differentiation is somewhat confounded by the frequency of EBV positivity across all subtypes [6], and the current classification systems recommend distinguishing EBV-negative from EBV-positive cases [7] [8]. The patient described in this case had sporadic BL, which accounts for 1% - 2% of adult lymphomas [9] and has a trimodal age-specific incidence, with peaks at 10 years (most frequent), 40 years (especially in males, who are affected three-to-four times more frequently than females), and 75 years [10].

In contrast to the mandibular masses frequently seen in endemic BL, sporadic BL has a high predilection for the gastrointestinal (GI) tract [4] [5] and frequently presents as a rapidly expanding intra-abdominal mass [11]. Indeed, the GI tract is themost common site of extranodal involvement across NHL [12], likely owing to the presence of Peyer’s patches throughout the GI submucosa [4]. BL most commonly arises in the ileocecal region, and presentation elsewhere in the GI tract is rare [13]. Likewise, primary GI lymphoma without evidence of nodal involvement is also uncommon, with an incidence of only 17% in a population-based registry [14].

Accordingly, the main GI complications of BL include bowel obstruction and intestinal perforation. Obstructions are classically due to mass effect and are often managed conservatively, due to the exquisite treatment sensitivity of BL and high likelihood of obstruction resolution with chemoimmunotherapy [15] [16]. Extensive resection should be avoided and surgical intervention is reserved for emergent situations such as perforation, recalcitrant GI bleeding, or persistent obstruction [16].

Bowel perforation is a feared complication that occurs in approximately 9% of lymphoma patients with GI involvement [17] [18]. In a retrospective series of over one thousand cases of biopsy-proven GI lymphoma, more than half (52.4%) of the patients had an aggressive B cell lymphoma, and the incidence of perforation in this subgroup was 10.9% [17]. Fifteen patients in the series were diagnosed with BL, one of whom experienced a perforation (incidence of 6.6%). Of 100 identified perforations, the small bowel was the most common site (59%), including 23 ileocecal events (23%). Though classically thought of as a complication of chemotherapy, essentially half (51%) of the perforation events in the series occurred prior to the receipt of systemic therapy. In patients who experienced perforation after treatment, the median time from initiation of therapy to perforation was 35 days, dispelling the prior notion that perforation is an early complication of therapy [17]. Still, the composite of gastrointestinal bleeding and perforation remains the second-leading cause of treatment-related mortality in BL after sepsis [19].

Intussusception is a rare complication of GI lymphoma. In general, intussusception is far more common in children and adolescents, with only 5% of intussusception cases occurring in adults [1]. While up to 20% of adult intussusception cases may be idiopathic, the vast majority (at least 80%) are secondary to a pathological lead point with an underlying tumor in roughly 75% of cases [1] [20]. Moreover, the clinical presentation of intussusception also differs between children and adults. The triad of emesis, hematochezia, and abdominal pain seenin children with intussusception is only present in 15% - 20% of adults who instead present with nausea and recurrent abdominal pain [21]. As abdominal ultrasound in adults is increasingly limited by body habitus and bowel gas, abdominal CT is widely regarded as the diagnostic modality of choice, with sensitivity of up to 100% in adults [22]. The classic imaging phenotype is a heterogenous “target” or “sausage-shaped” soft-tissue mass consisting of an outer intussuscipiens and central intussusceptum and isvirtually pathognomonic [23] [24] [25]. In contrast to children, attempts to reduce the intussuscepted bowel arenot recommended in adults due to the higher incidence of underlying malignancy and associated risks of perforation and peritoneal seeding. Thus, the management of adult intussusception remains primarily surgical except in the rare cases that resolve spontaneously [26] [27].

We identified 12 prior reports of adult intussusception secondary to BL indexed in MEDLINE (PubMed) [28]–[39]. The features of these cases are summarized in Table 1. The median age was 31 years, which is younger than the second age-specific incidence peak of 40 years [10]. However, the male-predominance of this incidence peak is reflected in the reported cases, with most patients (62%) being male. Abdominal pain was the most common presenting symptom (92%), with no patients reporting the traditional pediatric manifestation of hematochezia. The ileocolic region remains the common site of BL-associated intussusception, perhaps due to the abundance of Peyer’s patches in the terminal ileum [4].

Except for this patient’s case, all other instances of intussusception included in this cohort required either surgical resection (80%) or reduction (13%). A prior case series of adult intussusception from any cause reported a spontaneous resolution in 6 of 21 (29%) cases, though it is not clear how many of those cases were secondary to malignant lead points [40]. No patient with BL received upfront systemic chemotherapy as the primary means for addressing the intussusception. The course of our case, in conjunction with the outstanding response of BL to chemotherapy, prompts the question of whether a trial of conservative management should be considered for patients with BL-related intussusception who have reassuring abdominal exams.

Prognostication and outcome data for BL-associated intussusception remain scarce. The BL international prognostic index (BL-IPI) was reported in 2021 [3] and stratifies patients into low-, intermediate-, and high-risk disease categories based on four parameters, including age ≥ 40 years, Eastern Cooperative Oncology Group (ECOG) performance status ≥ 2, LDH more than three times the upper limit of normal for the reporting laboratory’s reference range, and the presence of CNS involvement. These risk categories correlate with three-year overall survival values of up to 99%, 85%, and 64%, respectively. Since publication of the BL-IPI, only two other cases of BL-associated intussusception have been reported in the literature [32] [33]. However, neither case documented the BL-IPI score or its components. To date, only four studies (including this one) reported patient outcome data, with follow up ranging from four months to three years. Though no mortality was reported in these cases, there is insufficient data to conclude whether intussusception is associated with worse overall survival in BL.

Ultimately, combination chemoimmunotherapy remains the standard-of-care for adults with BL. One of three regimens is recommended for first-line therapy: 1) R-CODOX-M alternating with IVAC; 2) R-hyper CVAD (rituximab with hyperfractionated cyclophosphamide, vincristine, doxorubicin, and dexamethasone typically alongside high-dose methotrexate and cytarabine); or 3) DA-EPO-CH-R (dose-adjusted etoposide, prednisone, vincristine, cyclophosphamide, and doxorubicin plus rituximab) [41] [42] [43] [44]. Patients with high-risk disease, bone marrow involvement, or CNS disease require high-intensity regimens, such as R-CODOX-M/IVAC (as in our patient) or R-hyper CVAD [42] [44]. Otherwise, a risk-adapted approach using DA-R-EPOCH is suitable for patients without CNS disease, as recent data suggests similar efficacy to R-CODOX-M/IVAC but with lower treatment-related toxicity [45]. While approximately 90% of children with BL are cured with highly dose-intensive chemotherapy, adults are more susceptible to the toxic effects of therapy and outcomes are less favorable [5]. Prognosis in adults varies based on several risk factors, including age, performance status, serum LDH, and CNS involvement, factors that comprise the BL-IPI [3]. Using this model, overall survival ranges from 59% - 96%, figures that compare favorably to real-world data from multiple centers [19] [46]. The significantly worse outcomes for patients with high-risk disease and the considerable toxicity associated with the requisite highly-dose intensive chemotherapy underscore the need to develop both safer and more efficacious therapies for adults with BL.

## 4. Conclusions

This case highlights the challenge of diagnosing intussusception in adults and the importance of considering intussusception as an etiology of recurrent abdominal pain. Although intussusception can occur at any age, the presentation and diagnostic approach differ between pediatric and adult populations. Early recognition of intussusception in adults with CT scan is crucial, considering that it is often associated with a malignant lead point. Sporadic BL is an uncommon entity and due to its predilection for the gastrointestinal tract, is a rare cause of adult intussusception that can be further complicated by bowel perforation and tumor lysis syndrome. Surgery is often required to resolve adult intussusception, though rare cases may revert with conservative management, as described here. Indeed, this case is the first report of BL-associated intussusception to resolve without interventional management.

Coupled with the observation that even high-risk (per the BL-IPI) cases of BL respond briskly to chemotherapy, this case raises the question of whether conservative management or expedited chemotherapy are safe options for patients with BL-related intussusception who exhibit reassuring abdominal exams. The rapid rise in LDH underscores the emergent nature of high-risk BL, and avoiding surgery allowed this patient to proceed promptly to high-dose chemotherapy for the widely disseminated and rapidly progressing disease. Due to the rarity of the presentation, however, there is currently insufficient data to conclude whether BL-associated intussusception portends a worse overall prognosis, especially when considering the concurrent risk of bowel perforation.

## Figures and Tables

**Table 1.** Reported features of adult intussusception secondary to Burkitt lymphoma.^a^

Category	Feature	Value^b^
Demographics	Age (years)	31 (21 - 53)
	Female sex	5/13 (38%)
	BL-IPI score^c^ [3]	2

Presenting symptoms	Abdominal pain	12/13 (92%)
	Nausea or vomiting	6/13 (46%)
	Constipation or obstipation	4/13 (31%)
	Hematochezia	0/13 (0%)

Location	Jejunal	4/13 (31%)
	Ileal	2/13 (15%)
	Ileocolic	5/13 (38%)
	Colonic	1/13 (8%)

Intervention^e^	Spontaneous resolution	1/15 (7%)
	Non-surgical reduction	2/15 (13%)
	Surgical resection	12/15 (80%)
	Systemic therapy^f^	0/15 (0%)

Outcomes	Mortality at 30-days^g^	0 (0%)

a Table includes data from the thirteen previously published cases and the case presented here.

b Data presented as median (range) or n (%).

c As the BL-IPI was developed in 2021 [3], no prior studies included a calculation of the prognostic index or reported the component data; thus, this BL-IPI score represents the index for the patient described in this manuscript alone.

e Two patients had recurrent intussusception requiring a second intervention.

f Indicates patients who received systemic therapy as the primary means for resolving the intussusception.

g Only four studies (including this manuscript) reported patient outcome data with follow up ranging from 4 months to 3 years.
